# Synthesis of 2D Nitrogen-Doped Mesoporous Carbon Catalyst for Oxygen Reduction Reaction

**DOI:** 10.3390/ma10020197

**Published:** 2017-02-17

**Authors:** Zhipeng Yu, Jinhua Piao, Zhenxing Liang

**Affiliations:** 1 Key Laboratory on Fuel Cell Technology of Guangdong Province, School of Chemistry and Chemical Engineering, South China University of Technology, Guangzhou 510641, China; yuzhipeng_scut@yeah.net; 2 School of Food Science and Engineering, South China University of Technology, Guangzhou 510641, China

**Keywords:** fuel cell, nitrogen-doped carbon, oxygen reduction reaction, 2D mesoporous carbon, 2D mesoporous silica

## Abstract

2D nitrogen-doped mesoporous carbon (NMC) is synthesized by using a mesoporous silica film as hard template, which is then investigated as a non-precious metal catalyst for the oxygen reduction reaction (ORR). The effect of the synthesis conditions on the silica template and carbon is extensively investigated. In this work, we employ dual templates—viz. graphene oxide and triblock copolymer F127—to control the textural features of a 2D silica film. The silica is then used as a template to direct the synthesis of a 2D nitrogen-doped mesoporous carbon. The resultant nitrogen-doped mesoporous carbon is characterized by transmission electron microscopy (TEM), nitrogen ad/desorption isotherms, X-ray photoelectron spectroscopy (XPS), cyclic voltammetry (CV), and rotating disk electrode measurements (RDE). The electrochemical test reveals that the obtained 2D-film carbon catalyst yields a highly electrochemically active surface area and superior electrocatalytic activity for the ORR compared to the 3D-particle. The superior activity can be firstly attributed to the difference in the specific surface area of the two catalysts. More importantly, the 2D-film morphology makes more active sites accessible to the reactive species, resulting in a much higher utilization efficiency and consequently better activity. Finally, it is noted that all the carbon catalysts exhibit a higher ORR activity than a commercial Pt catalyst, and are promising for use in fuel cells.

## 1. Introduction

The oxygen reduction reaction (ORR) is one of the most important reactions in energy conversion devices such as fuel cells and metal-air batteries [1,2]. Pt has been the mostly widely used and effective catalyst for the ORR [3,4,5]; however, the source scarcity and high cost hinder the large-scale application of fuel cells [6,7]. Hence, enormous efforts have been devoted to developing alternative non-precious-metal catalysts [8,9,10,11,12,13].

Nanostructured heteroatom-doped carbon has attracted intensive attention in the past decades [14,15,16,17,18,19,20,21,22,23]. Among them, 2D nitrogen-doped carbon is an ideal candidate as an electrocatalyst for the ORR due to its unique features [24,25,26,27]. Feng et al. [28] synthesized 2D graphene-based carbon nitride nanosheets, the high specific surface area of which favors a dense assembly of the active sites. Mukerjee et al. [29] synthesized a nitrogen-doped graphene through a two-step solution-based procedure which presents a superior ORR activity. Yu et al. [30] used biomass as the precursor to synthesize nitrogen-doped nanoporous carbon nanosheets. The resultant carbon shows a high specific surface area and enriched micropores, yielding an excellent catalytic activity for the ORR.

Graphene and graphitic carbon nitride have been widely used as a template to direct the synthesis of other 2D materials [31,32,33,34,35]. Wang et al. [31] developed nitrogen-doped nanoporous carbon/graphene nano-sandwiches by using graphene oxide (GO) as a template, which exhibits a high onset potential of the ORR. Zhang et al. [32] employed g-C_3_N_4_ as a template to synthesize nitrogen-doped porous carbon nanosheets, which showed a superior ORR performance in alkaline media.

In our previous work, we developed a co-operative assembly method with the dual templates of GO and tri-block copolymer P123 to synthesize an ultrathin 2D semi-ordered mesoporous silica film [36]. In this work, we employed F127—another tri-block copolymer—to further control the textural features of the 2D silica film. Then, the silica was used as a template to direct the synthesis of the 2D nitrogen-doped mesoporous carbon. The resultant nitrogen-doped mesoporous carbon was extensively investigated by transmission electron microscopy (TEM), nitrogen ad/desorption isotherms, X-ray photoelectron spectroscopy (XPS), cyclic voltammetry (CV), and rotating disk electrode measurements (RDE). It was found that the synthesized carbon catalyst yielded a much better electrocatalytic activity than its Pt counterpart.

## 2. Results and Discussion

### 2.1. 2D Mesoporous Silica Template

**Figure 1:**
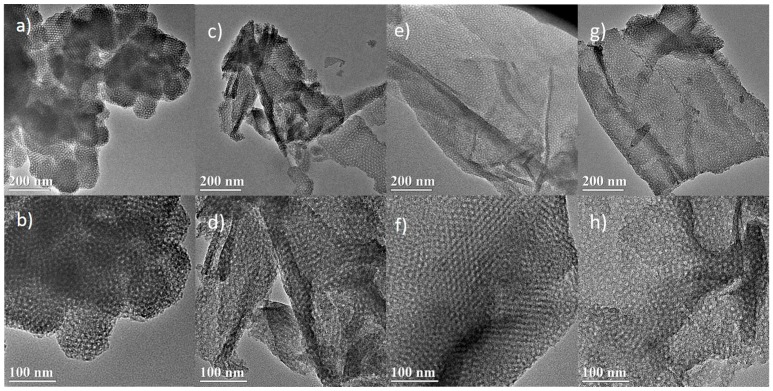
TEM images of the SiO_2_/GO-*x* (GO: graphene oxide; *x:* GO concentration): (**a**,**b**) *x* = 0; (**c**,**d**) *x* = 2.32; (**e**,**f**) *x* = 4.64; (**g**,**h**) *x* = 9.28.

Figure 1 shows TEM images of the synthesized silicas by using F127 and GO as the dual template. The samples are referred to as SiO_2_/GO-*x* (*x*: GO concentration). Figure 1a,b reveals that the silica of SiO_2_/GO-0 synthesized with the sole template (F127) exhibits a highly-ordered 3D close-packed cage-type mesoporous structure—namely KIT-5 [37]. When GO is added as a co-template, the resultant silica evolves from the aforementioned 3D particles to a 2D film, and considerable change occurs in both morphology and characteristic dimension. Figure 1c–h shows that the silica film gradually develops with increasing GO concentration. For example, Figure 1e shows that the resultant silica film is complete and thin at 4.64 mg·mL^−1^. Further increase in the GO concentration yields some aggregation of the silica film, as seen in Figure 1g. Besides, cage-type mesopores are well recognized in the resultant silicas, with diameter in the range of 4–6 nm. It has been proposed that the formation of the 2D mesoporous silica film proceeds by the so-called cooperative assembly mechanism, as discussed in our previous publication [36]. Briefly, the 2D mesoporous structure is generated with the aid of dual templates. The mesopores are a negative replica of the template F127, and the 2D morphology inherits that of the GO template. The final structure relies on the cooperative assembly among the silica precursor, F127, and GO.

**Figure 2:**
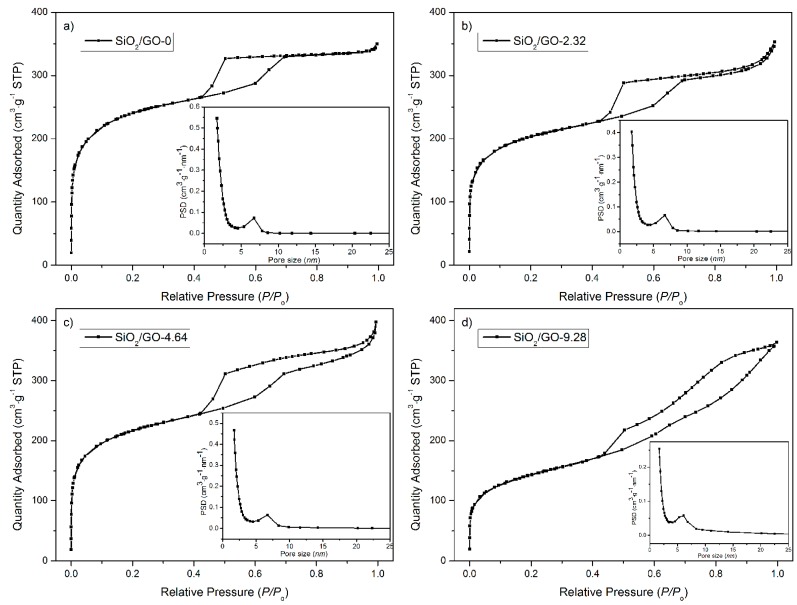
Nitrogen ad/desorption isotherms of the synthesized silicas SiO_2_/GO-*x*: (**a**) *x* = 0; (**b**) *x* = 2.32; (**c**) *x* = 4.64; (**d**) *x* = 9.28.

Figure 2 shows the corresponding nitrogen ad/desorption isotherms, from which the pore features are extracted and listed in Table S1. SiO_2_/GO-0 shows a typical type-IV isotherm with an H2 hysteresis loop, confirming its ordered mesoporous structure with uniform cage-type pores [38,39]. Similarly, when GO is used as a co-template, the resultant silicas show the type-IV isotherms and thereby mesoporous structure. It is, however, noted that the hysteresis loop gradually evolves from H2 to H3 with increasing GO concentration. It is acknowledged that the H3 hysteresis loop originates from aggregates of plate-like particles [40]. It is thus inferred that the aggregation of the silica film occurs when the GO concentration is sufficiently high (viz. 9.28 mg·mL^−1^), which agrees well with the aforementioned TEM observations. The calculation of the specific surface area further confirms this analysis. The specific surface area is 708, 756, and 504 m^2^·g^−1^ for SiO_2_/GO-2.32, SiO_2_/GO-4.64, and SiO_2_/GO-9.28, respectively. The sharp decrease in the specific surface area of SiO_2_/GO-9.28 intuitively suggests a serious aggregation in the silica film.

Afterwards, the resultant silicas were used as the template to synthesize nitrogen-doped mesoporous carbon, which is referred to as NMC-SiO_2_/GO-*x*.

### 2.2. 2D Mesoporous Carbon

**Figure 3:**
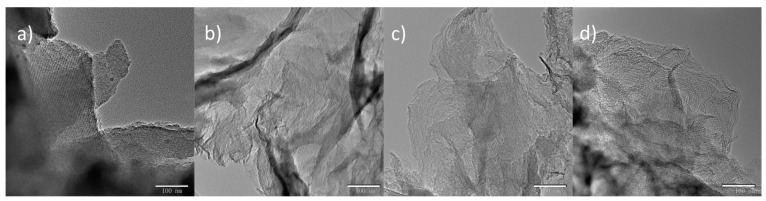
TEM images of the nitrogen-doped mesoporous carbon (NMC)-SiO_2_/GO-*x*: (**a**) *x* = 0; (**b**) *x* = 2.32; (**c**) *x* = 4.64; (**d**) *x* = 9.28.

Figure 3 shows the TEM images of the synthesized NMC by using the above silica templates. Figure 3a shows that NMC-SiO_2_/GO-0 follows the negative replica of SiO_2_/GO-0, which is a bulky particle associated with an ordered mesoporous structure (Figure S1). In comparison, by using 2D silica film, the other three carbons are 2D film embedded with mesopores, as seen in Figure 3b–d.

**Figure 4:**
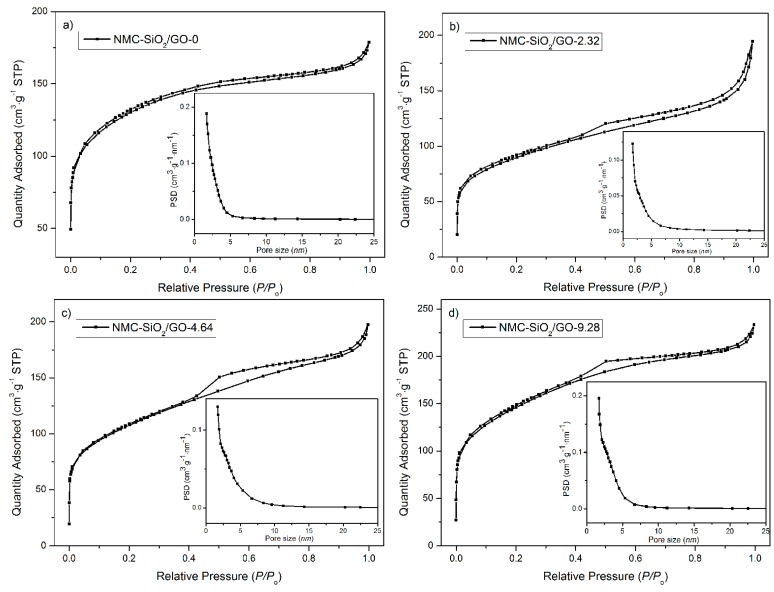
Nitrogen ad/desorption isotherms of the synthesized carbon (NMC)-SiO_2_/GO-*x*: (**a**) *x* = 0; (**b**) *x* = 2.32; (**c**) *x* = 4.64; (**d**) *x* = 9.28.

Figure 4 shows the corresponding nitrogen ad/desorption isotherms. It is seen that all the curves display a type-IV isotherm, indicating their mesoporous structure. The specific surface area (see Table 1) follows the order: NMC-SiO_2_/GO-2.32 < NMC-SiO_2_/GO-4.64 < NMC-SiO_2_/GO-0 < NMC-SiO_2_/GO-9.28. It is understandable that this result will yield an effect on the electrochemical behavior (vide infra).

Besides the structure, the content of dopant nitrogen is one of the parameters that most determines the electrocatalytic activity. To clarify the contribution, the surface composition was characterized by XPS (see Figure S2), and the elemental content is listed in Table 2. It is seen that the nitrogen content is similar ca. 3.80 at %, except that NMC-SiO_2_/GO-9.28 shows a slightly higher content.

### 2.3. Interfacial Electrochemistry of Carbon

**Figure 5:**
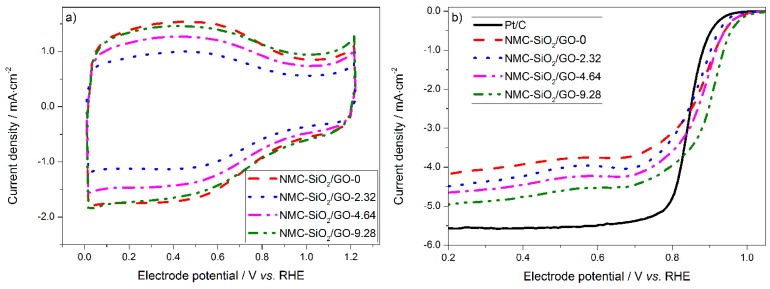
(**a**) Cyclic voltammograms in Ar-saturated 0.10 M KOH solution; (**b**) the corresponding oxygen reduction reaction (ORR) polarization curves in O_2_-saturated 0.10 M KOH solution. RHE: reversible hydrogen electrode.

Figure 5 shows the CV curves and the corresponding ORR polarization curves of the carbon materials in 0.10 M KOH solution. Figure 5a shows that all of the curves are similar in shape with large capacitive currents, which can be attributed to their high specific surface area. The capacitive current follows the order: NMC-SiO_2_/GO-2.32 < NMC-SiO_2_/GO-4.64 < NMC-SiO_2_/GO-9.28 ~ NMC-SiO_2_/GO-0, which agrees well with the change in the specific surface area (vide supra). A broad and electrochemically-reversible wave was observed in the potential range of 0–0.9 V, which is acknowledged to originate from the adsorption of OH^−^ on the carbon surface [41,42]. In addition, the redox peak associated with iron-containing species is not observed, which indicates that the amount of electrochemically active Fe on the carbon surface is negligible [43].

Figure 5b shows the ORR polarization curves in the O_2_-saturated 0.10 M KOH solution. The electrocatalytic activity of the carbon catalysts follows the order of NMC-SiO_2_/GO-0 < NMC-SiO_2_/GO-2.32 < NMC-SiO_2_/GO-4.64 < NMC-SiO_2_/GO-9.28. To rationalize this result, both the active sites and specific surface area need to be considered. First, the active sites were found to be the nitrogen-activated carbon atoms. Both the content and the chemical state of the dopant nitrogen were found to be similar for the four carbon catalysts (Figure S3 and Table S2). Therefore, the density of active sites is similar, which cannot explain the large difference in the electrocatalytic activity. Second, the specific surface area and the consequent electrochemically active surface area vary considerably for the four carbon catalysts. This should make a remarkable contribution in the difference in the activity. In addition, it is noted that the current density at low electrode potentials of the 2D carbon catalyst is much larger than that of the 3D carbon particle. In our previous work, it was found that the active sites in the 2D carbon film are more accessible to the electrolyte and the reactive species [36]. This morphological feature results in a much higher utilization efficiency of the active sites. In comparison, for the 3D carbon particle, most active sites are anchored on the inner walls of the mesopores. Therefore, the reactive species must travel through the long-range mesopores before reaching those active sites, resulting in a poor utilization efficiency. This issue will degrade the kinetics, which becomes more pronounced at large current densities. Finally, it is noted that all the carbon catalysts exhibit a higher ORR activity than does the commercial Pt catalyst, and are promising for use as Pt alternatives in fuel cells.

## 3. Materials and Methods 

### 3.1. Materials Preparation

The aqueous dispersion of graphene oxide (2.6 wt %) was gifted by Ningbo Institute of Material Technology and Engineering, and was directly used as obtained.

The mesoporous Fm3m silica, namely KIT-5, was synthesized as reported elsewhere [37]. Typically, 2.5 g tri-block copolymer, EO_106_PO_70_EO_106_ (Pluronic F127, BASF, Hamburg, Germany) was dissolved in 120 g of deionized water and 5.3 g of HCl (37 wt %). Then, 12.0 g tetraethyl orthosilicate (TEOS, 99%) was stirred for 24 h at 45 °C and subsequently hydrothermally treated at 100 °C for 24 h. The product was filtered, dried, and ground. Finally, the template was removed by calcination in air at 550 °C for 6 h. The obtained silica was referred to as SiO_2_/GO-0. The same process was repeated to synthesize other silicas in the presence of GO. The synthesis conditions are listed in Table S3.

The nitrogen-doped mesoporous carbons (NMCs) were synthesized via a nanocasting method using the above-mentioned silica as the template [44]. First, 3.0 g silica was dispersed in an ethanol solution (20.0 mL ethanol + 20.0 mL deionized water). Second, phenanthroline was dissolved in 10.0 mL ethanol and mixed with a FeCl_2_ aqueous solution. The molar ratio of iron to phenanthroline was 1:3 to ensure complete coordination. Then, the above two mixtures were mixed and sonicated for 6 h. After the solvent was evaporated, the resultant powders were pyrolyzed at 900 °C for 3 h in argon (99.999%). Finally, the NMC catalysts were obtained by removing the silica template and Fe species. The template was removed by refluxing the powders in 10 M NaOH at 120 °C for 24 h, and the iron species was leached out by boiling the powders in 0.10 M HClO_4_ at 80 °C for 24 h.

The samples were referred to as NMC-SiO_2_/GO-0, NMC-SiO_2_/GO-2.32, NMC-SiO_2_/GO-4.64, and NMC-SiO_2_/GO-9.28, which correspond to the template of SiO_2_/GO-0, SiO_2_/GO-2.32, SiO_2_/GO-4.64, and SiO_2_/GO-9.28, respectively.

### 3.2. Physical Characterizations

Transmission electron microscopy (TEM) was performed on a FEI Tecnai G2 F20 S-TWIN (Hillsboro, OR, USA) operated at 200 kV. X-ray photoelectron spectroscopy (XPS, Physical Electronics PHI 5600, Chanhassen, MN, USA) measurement was carried out with a multi-technique system using an Al monochromatic X-ray at a power of 350 W. Nitrogen adsorption/desorption isotherms were measured at 77 K using a Micromeritics TriStar II 3020 analyzer (Norcross, GA, USA). Before the measurements, the samples were outgassed for 12 h in the degas port of the adsorption apparatus, at 473 K for the calcined samples. The total surface area was analyzed with the well-established Brunauer-Emmett-Teller (BET) method, and the pore size distribution was calculated on the basis of adsorption branches of nitrogen isotherms using the Barrett-Joyner-Halenda (BJH) method. 

### 3.3. Electrochemical Characterization

The electrochemical behavior of the catalyst was characterized by the cyclic voltammetry (CV) and linear sweeping voltammetry (LSV) using a three-electrode cell with an electrochemical work station Zennium (Zahner, Germany) at room temperature (25 °C). A gold wire and a double-junction Ag/AgCl reference electrode were used as the counter and reference electrodes, respectively. The working electrode was a rotating disk electrode (RDE, glassy carbon disk: 5.0 mm in diameter). The thin-film electrode on the disk was prepared as follows: 10.0 mg of the catalyst was dispersed in 1.0 mL Nafion/ethanol (0.84 wt % Nafion) by sonication for 60 min. Then, 10 μL of the dispersion was transferred onto the glassy carbon disk by using a pipette, yielding the catalyst loading of 0.50 mg·cm^−2^. The ORR activity of the Pt/C catalyst (HiSPEC4000, Johnson Matthey, London, UK) with the metal loading of 20 μg·cm^−2^ was collected for comparison.

The electrolyte solution (0.10 M KOH) was first bubbled with argon for 60 min. Then, a CV test was conducted at 20 mV·s^−1^ in the potential range between 0 and 1.23 V (vs. reversible hydrogen electrode, RHE) for 20 cycles. If not specified, the LSV curve was collected by scanning the disk potential from 1.2 down to 0 V at 5 mV·s^−1^ in the oxygen-saturated electrolyte solution under 1600 rpm, from which the ORR polarization curve was extracted by subtracting the capacitive current.

## 4. Conclusions

In this work, 2D nitrogen-doped mesoporous carbon (NMC) was developed as the catalyst for the oxygen reduction reaction (ORR). Physico-chemical characterizations reveal that the resultant carbon features 2D morphology, mesoporous structure, and high specific surface area. Electrochemical testing showed that the carbon catalyst yields a highly electrochemically active surface area and superior electrocatalytic activity for the ORR compared to the 3D-particle one. It was found that the superior activity of the 2D-NMC catalyst is attributed to its higher specific surface area and utilization efficiency of the active sites. The above findings demonstrate the importance of the nanostructure in favoring the electrocatalysis from the view of material engineering.

## Figures and Tables

**Table 1:** Pore features of the synthesized carbon.

Sample	A_BET_/m^2^·g^−1^	D_BJH_/nm	V/cm^3^·g^−1^
NMC-SiO_2_/GO-0	453	3.0	0.26
NMC-SiO_2_/GO-2.32	314	4.5	0.31
NMC-SiO_2_/GO-4.64	377	3.8	0.32
NMC-SiO_2_/GO-9.28	513	3.3	0.37

**Table 2:** Elemental composition (at %) of the synthesized carbon.

Sample	C	N	O	N:C
NMC-SiO_2_/GO-0	85.78	3.86	10.36	0.045
NMC-SiO_2_/GO-2.32	86.70	3.79	9.51	0.044
NMC-SiO_2_/GO-4.64	83.08	3.72	13.20	0.045
NMC-SiO_2_/GO-9.28	84.24	4.19	11.57	0.050

## Supplementary Materials

The following are available online at www.mdpi.com/1996-1944/10/2/197/s1. Figure S1: TEM image of the NMC-SiO_2_/GO-0, Figure S2: XPS survey spectra of carbon, Figure S3: N 1s peak and the peak fitting results of the following materials: (a) NMC-SiO_2_/GO-0; (b) NMC-SiO_2_/GO-2.32; (c) NMC-SiO_2_/GO-4.64; (d) NMC-SiO_2_/GO-9.28, Figure S4: C 1s peak and the peak fitting results of the following materials: (a) NMC-SiO_2_/GO-0; (b) NMC-SiO_2_/GO-2.32; (c) NMC-SiO_2_/GO-4.64; (d) NMC-SiO_2_/GO-9.28, Table S1: Pore features of the synthesized silica materials, Table S2: Content of each nitrogen component (%) of the synthesized carbon, Table S3. The synthesis conditions of silica.
