# Supplementary material for: Synthesis of 2D Nitrogen-Doped Mesoporous Carbon Catalyst for Oxygen Reduction Reaction
Source: Materials (Basel). 2017 Feb 17;10(2):197. doi: 10.3390/ma10020197 (PMC5459128; doi:10.3390/ma10020197)
Supplement: Supplementary file 1 [file materials-10-00197-s001.pdf]

Supplementary Information

Synthesis of 2D Mesoporous Nitrogen-doped Carbon Catalyst for Oxygen Reduction Reaction

Zhi-peng Yu[a], Jin-hua Piao [b*], Zhen-xing Liang[a*]

[a]Key Laboratory on Fuel Cell Technology of Guangdong Province, School of Chemistry and Chemical Engineering, South China University of Technology, Guangzhou 510641, P.R. China

[b]School of Food Science and Engineering, South China University of Technology, Guangzhou 510641, P.R. China

Corresponding authors: Tel.: +86 20 87113849, E-mail: jhpiao@scut.edu.cn (J.H. Piao); Tel.: +86 20 87113584, E-mail: zliang@scut.edu.cn (Z.X. Liang).

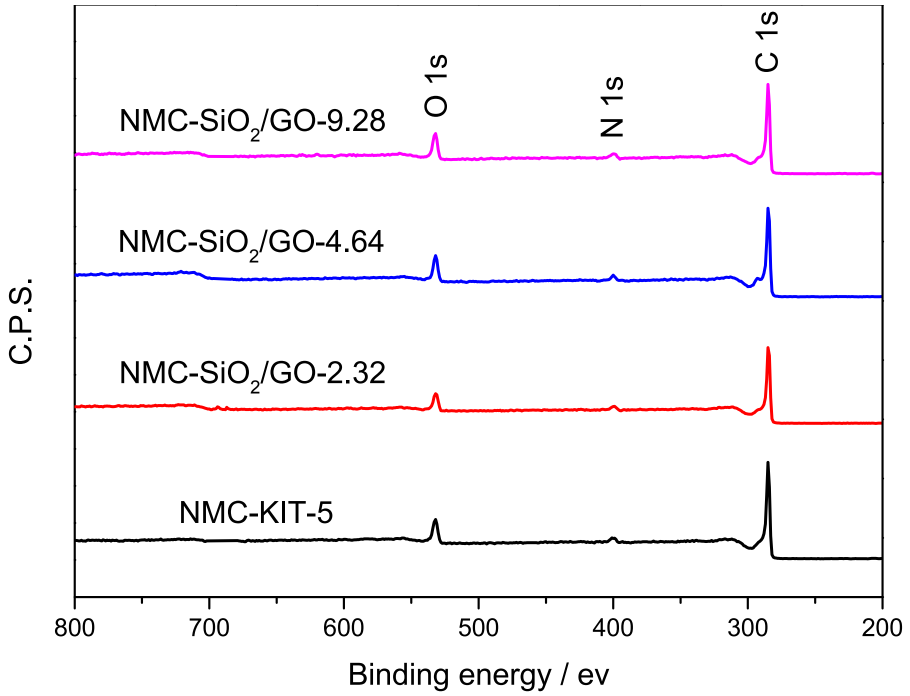

Figure S1. X-ray photoelectron spectroscopy (XPS) survey spectra of carbon. NMC: nitrogen-doped mesoporous carbon.

The high-resolution spectra were collected to further interrogate the chemical state of the dopant nitrogen. Figure S2 presents the experimental curve and fitting result. N 1s is deconvoluted to three peaks, which correspond to pyridinic-, graphitic-, and oxide-nitrogen, respectively. Based on the curve fitting, the content of each species is listed in Table S2. It is seen that the dopant nitrogen is mainly composed of pyridinic- and graphitic-nitrogen.

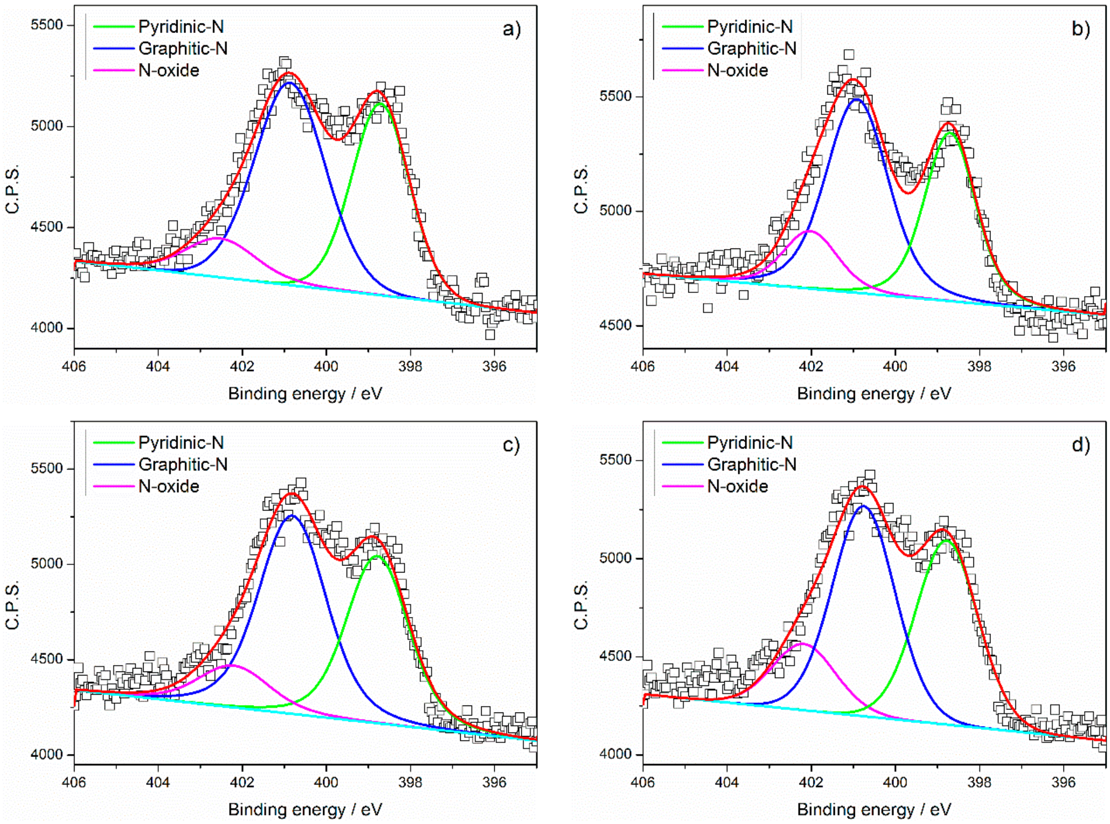

Figure S2. N 1s peak and the peak fitting results of the following materials: (**a**) NMC-SiO_2/GO-0; (**b**) NMC-SiO_2/GO-2.32; (**c**) NMC-SiO_2/GO-4.64; (**d**) NMC-SiO_2/GO-9.28.

The high-resolution spectra of carbon were collected to differentiate the content of the nitrogen-activated carbon atoms, which act as the active site for the oxygen reduction reaction (ORR). Figure S3 presents the experimental curve and fitting result. The deconvolution result indicates that the content of nitrogen-activated carbon atoms is similar for the four carbon catalysts, which is in the range of 10%–11%. Therefore, the difference in the electrocatalytic activity cannot be explained by the density of the active sites on the surface of the catalysts.

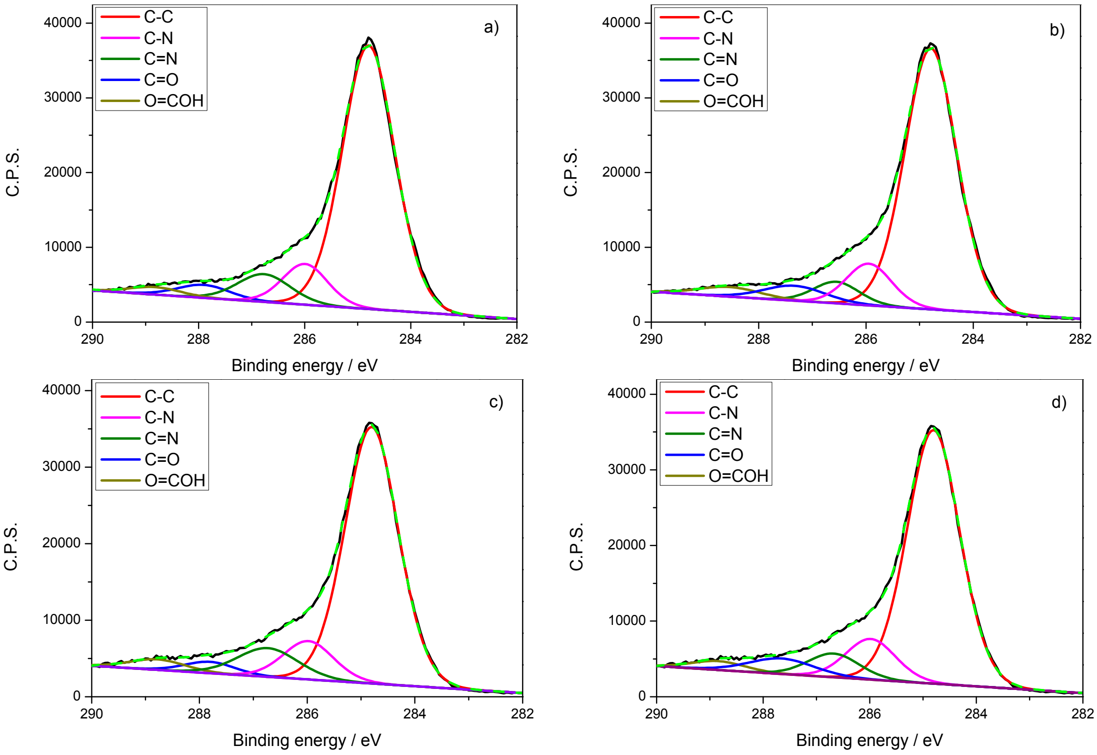

Figure S3. C 1s peak and the peak fitting results of the following materials: (**a**) NMC-SiO_2/GO-0; (**b**) NMC-SiO_2/GO-2.32; (**c**) NMC-SiO_2/GO-4.64; (**d**) NMC-SiO_2/GO-9.28.

Table S1. Pore features of the synthesized silica materials.

Sample	$A_{BET}/m^2 \cdot g^{-1}$	D_{BJH}/nm	$V/cm^3 \cdot g^{-1}$
SiO_2/GO-0	838	2.9	0.59
SiO_2/GO-2.32	708	3.6	0.56
SiO_2/GO-4.64	756	3.6	0.66
SiO_2/GO-9.28	504	4.8	0.61

Table S2. Content of each nitrogen component (%) of the synthesized carbon.

Sample	Pyridinic-N	Graphitic-N	O-N
NMC-SiO_2/GO-0	39.07	50.69	10.24
NMC-SiO_2/GO-2.32	36.09	50.71	13.21
NMC-SiO_2/GO-4.64	38.79	50.09	11.12
NMC-SiO_2/GO-9.28	38.61	51.09	10.29

Table S3. The synthesis conditions of silica.

Sample	Concentration	
	**F127/mmol·L^{-1}**	**GO/mg·mL^{-1}**
SiO_2/GO-0	1.59	0
SiO_2/GO-2.32	1.59	2.32
SiO_2/GO-4.64	1.59	4.64
SiO_2/GO-9.28	1.59	9.28
